# In-Vitro dual inhibition of protein glycation, and oxidation by some Arabian plants

**DOI:** 10.1186/s12906-016-1225-7

**Published:** 2016-08-05

**Authors:** Maqsood A. Siddiqui, Saima Rasheed, Quaiser Saquib, Abdulaziz A. Al-Khedhairy, Mansour S. Al-Said, Javed Musarrat, Muhammad Iqbal Choudhary

**Affiliations:** 1 Department of Zoology, College of Science, King Saud University, P. O. Box. 2455, Riyadh, 11451 Saudi Arabia; 2 H.E.J. Research Institute of Chemistry, International Center for Chemical and Biological Sciences, University of Karachi, Karachi, 75270 Pakistan; 3 A. R. Al-Jeraisy Chair for DNA Research, Zoology Department, College of Science, King Saud University, P. O. Box. 2455, Riyadh, 11451 Saudi Arabia; 4 Department of Agriculture Microbiology, Faculty of Agriculture Sciences, AMU, Aligarh, 202002 India; 5 Departments of Pharmacognosy, College of Pharmacy, King Saud University, P. O. Box. 2455, Riyadh, 11451 Saudi Arabia

**Keywords:** Arabian medicinal plants, Diabetes, Advanced glycation end products (AGEs), Antioxidant, *Glycyrrhiza glabra* L., *Rosa indica* L., *Sida cordifolia* L.

## Abstract

**Background:**

Diabetes mellitus is a metabolic disorder of epidemic proportion, projected to become the major cause of morbidity and mortality in the world in future. Despite extensive research in understanding this disease at molecular level, and the discovery of new drugs, diabetes and its complications remain largely untreated. Many of the late diabetic complications are associated with the glycation of proteins in the body. Natural flora has long been a rich source for therapeutic agents, especially against diabetes. The present study deals with the anti-glycation properties of some medicinally important plants of Arabian region.

**Methods:**

Twenty-six medicinal plants, commonly found in different regions of Arabian Peninsula, were evaluated for their protein anti-glycation activity by using BSA-MG glycation assay in-vitro. The extracts were incubated with BSA and MG at 37 °C for 9 days, each sample was then examined for the presence of fluorescence (λex 330 nm, and λem 420 nm), which represent the extent of protein glycation. Antioxidant activity was evaluated by using 1,1-diphenyl- 2-picrylhydrazyl (DPPH), iron chelation, and superoxide radical scavenging asaays.

**Results:**

The data revealed that out of 26 medicinal plants, five plants *viz. Sida cordifolia*, *Plumbago zeylanica*, *Tribulus terrestris*, *Glycyrrhiza glabra*, and *Rosa indica* were active against the in-vitro protein glycation with IC_50_ values between 0.408- 1.690 mg/mL. Among the active plants, *Glycyrrhiza glabra* L. was found to be the most potent (IC_50_ = 0.408 ± 0.027 mg/mL), followed by *Rosa indica* (IC_50_ = 0.596 ± 0.0179 mg/mL), and *Sida cordifolia* L. (IC_50_ = 0.63 ± 0.009 mg/mL). The antioxidant potential of these plant extracts were also determined by using DPPH (2,2-diphenyl-1-picrylhydrazyl), iron chelation, and superoxide anion radical scavenging assays. Among five plants, *Sida cordifolia* exhibited a potent anti-oxidant activity in both DPPH and superoxide anion radical scavenging assays (IC_50_ = 0.005 ± 0.0004, and 0.078 ± 0.002 mg/mL, respectively), followed by *Rosa indica* (IC_50_ = 0.023 ± 0.0005 and 0.141 ± 0.003 mg/mL, respectively).

**Conclusions:**

Protein glycation in hyperglycemic conditions involve oxidative changes. Therefore dual inhibition of protein glycation and oxidation are desirable properties in any test substance investigated for therapeutic purposes.

## Background

Diabetes mellitus (DM) is an impending public health challenge of the present century [1]. It affects over 387 million people globally, and this number is projected to increase to 592 million by 2035. DM is currently the fourth leading cause of mortality in the world. It has also emerged as a major socioeconomic burden for developing countries [2]. In last three decades, extensive research has been conducted on glycation and anti-glycation processes in diabetes, based on the fact that the hyperglycemic condition or excess glucose in blood leads to the binding of free sugars with bio-molecules [3–5]. Glycation is a spontaneous, non-enzymatic reaction between biomolecules (proteins, lipids, and DNA) and reducing sugars (such as glucose, fructose, and ribose), resulting in the formation of advanced glycation endproducts (AGEs) [6–8]. The accelerated process of proteins glycation has been identified as a marker, as well as a core reason for the onset of many diabetic complications, affecting the eyes, blood vessels, kidneys, skin, *etc*. [9, 10]. Oxidative reactions are known to be involved in the protein glycation cascade. Most importantly, AGEs, *via* their receptors (RAGEs), inactivate the enzymes and promote the formation of reactive oxygen species (ROS). It is suggested that the generation of oxygen free radicals by glycation of biomolecules is one of the major biochemical pathways of oxidative tissue damage in diabetes. Search for agents with dual inhibitory effects, *i.e*. antioxidant and anti-glycation, is therefore a valid approach towards the treatment of complications resulting from non-enzymatic glycation reaction [11, 12]. Although extensive research has been conducted on various classes of glycation inhibitors, but none has reached to the clinical use. Therefore, there is an urgent need to identify the agents which inhibit or reverse the complex reactions of protein glycation, and oxidation.

Evidences about the anti-diabetic properties of medicinal plants have been continuously reported. During the last two decades, we have been focusing on bioactive natural products. This has led to the identification of several classes of safe and effective lead molecules [12–16]. Information on dual inhibition pattern (anti-glycation and antioxidant activity) of traditional Arabian medicinal plants is scarce. So far no large-scale systematic study of the anti-glycation activity of medicinal herbs has been conducted. Therefore, the present study was designed to identify new and effective inhibitors of protein glycation during hyperglycemia from medicinal plants of Arabian region. We evaluated 26 medicinal plants, commonly found in different regions of Arabian Peninsula. These plants are used in herbal medicines for the treatment of different diseases, including diabetes.

## Methods

### Chemicals

Bovine serum albumin (BSA), and ethanol was purchased from Merck Marker Pvt. Ltd. (Germany), methylglyoxal (MG) (40 % aqueous solution), 2,2-diphenyl-1-picrylhydrazyl (DPPH), iron chloride, ferrozine, *β*-nicotanamide adenine dinucleotide (NADH), nitro blue tetrazolium (NBT), phenazine methosulphate (PMS), Quercetin (purity: ≥95.0 %), Gallic acid (purity: ≥98.0 %), and rutin (purity: ≥90 %) were from Sigma Aldrich (Japan). Sodium azide (NaN_3_), disodium hydrogen phosphate (Na_2_HPO_4_), and sodium dihydrogen phosphate (NaH_2_PO_4_) were obtained from Scharlau Chemie, S. A. (Spain), while dimethyl sulphoxide (DMSO) was acquired from Fischer Scientific (UK).

### Plant material

All plant samples were collected from different regions of Arabian Peninsula. Different parts of these plants (such as leaves, flowers, stems, or roots; Table 1) were separately processed for the preparation of crude extracts. Plants were identified by taxonomist at the Department of Botany, University of Karachi, Karachi, Pakistan (Herbarium voucher numbers are mentioned in supporting information). The samples were air-dried, protecting from sunlight, and powdered. These powdered samples were then stored at room temperature.

### Preparation of the crude extracts of medicinally important plants

Crude extracts were prepared by extracting different powdered parts of the plants (1 Kg) in 3 L distilled methanol. In brief, the extracts were obtained by triple soaking in methanol for 3 days (at room temperature) and the solvent was evaporated under reduced pressure. The crude extracts were then freeze dried, and the extracts were solublized in DMSO and used for the in-vitro experiments.

### In-vitro anti-glycation assay

The reaction was performed in triplicate, and in such a way that in 200 *μ*L solution, the final concentration of BSA was 10 mg/mL, methylglyoxal was 14 mM, and test extracts (dissolved in DMSO; final concentration 10 %) were 2 mg/mL. Solution of methylglyoxal and BSA were prepared in phosphate buffer (0.1 M, pH 7.4, containing 3 mM sodium azide as antimicrobial agent). Briefly, the 200 *μ*L of reaction mixture comprised of BSA (50 *μ*L), methylglyoxal (50 *μ*L), test extracts (20 *μ*L), and phosphate buffer (80 *μ*L), while in the negative control wells, 20 *μ*L of DMSO (final concentration 10 %) was added instead of test extracts. It was then incubated at 37 °C for 9 days (under sterile conditions). After incubation, each sample was examined for the development of fluorescence (λex 330 nm and λem 420 nm), against blank on a microtitre plate reader (SpectraMax M5, Molecular Devices, CA, USA) [17]. Rutin was used as positive control. The percent inhibition of each extract was calculated by using the following formula:

$$ \%\ \mathrm{Inhibition} = \left(1 - \mathrm{Fluorescence}\ \mathrm{of}\ \mathrm{test}\ \mathrm{sample}/\ \mathrm{Fluorescence}\ \mathrm{of}\ \mathrm{the}\ \mathrm{control}\right) \times 100 $$

### In-vitro antioxidant activities

#### DPPH Free radical scavenging assay

Solution of DPPH (0.3 mM) was prepared in ethanol, while different concentrations of the test extracts were prepared in DMSO. In each well of 96-wells plate, 5 *μ*L of the test extracts and 95 *μ*L of DPPH solution were added, and the pre-read (absorbance) was recorded at 515 nm. The reaction was then incubated for 30 min at 37 °C. Plate was shaken for 1 min for thorough mixing and the change in absorbance was recorded at 515 nm on microplate-reader (SpectraMax M5, Molecular Devices, CA, USA) [18]. Gallic acid was used as a positive control. The percentage of DPPH radical scavenging was calculated by using following formula:

$$ \%\ \mathrm{R}\mathrm{S}\mathrm{A} = 100 - \left(\varDelta \mathrm{A}\ \mathrm{S}\mathrm{ample}\ /\varDelta \mathrm{A}\ \mathrm{Control}\right)\ \mathrm{x}100 $$

(Where RSA = radical scavenging activity and ΔA = change in absorbance)

### Iron chelation assay

The Fe^2+^-chelating ability was determined according to the method of Koncic et al. with slight modifications [19]. In this assay, the concentration of Fe^2+^ ion was measured through the formation of ferrous ion–ferrozine complex. Plant extracts, dissolved in DMSO (2 mg/mL, 5 *μ*L), was mixed with 0.3 mM FeCl_2_ (35 *μ*L) and 0.5 mM ferrozine (60 *μ*L). Ferrozine reacted with the divalent iron resulting in the formation of stable violet colored complex (soluble in water). The mixture was shaken and left at room temperature for 10 min. The change in the absorbance of the resulting mixture was measured at 562 nm by using SpectraMax M5 (Molecular Devices, CA, USA). Disodium EDTA was used as a reference compound.

### Superoxide anion radical scavenging assay

The reaction mixture contained 10 *μ*L of crude plant extracts (2 mg/mL; dissolved in DMSO), 90 *μ*L of phosphate buffer (0.1 M; pH 7.4), 40 *μ*L of (0.2 mM) *β*-nicotanamide adenine dinucleotide (NADH), and 40 *μ*L of (0.081 mM) nitro blue tetrazolium (NBT). The reaction was initiated by the addition of 20 *μ*L of (0.008 mM) phenazine methosulphate (PMS). The solutions of NADH, NBT and PMS were prepared in phosphate buffer (0.1 M; pH 7.4) [20]. The formation of superoxide was monitored by measuring the absorbance of the blue formazan dye after 5 min at 560 nm by using microtitre plate reader (SpectraMax M5, Molecular Devices, CA, USA). Quercetin was used as a positive control.

### Statistical analysis

The results were analyzed by using SoftMax Pro Software (Molecular Devices, CA, USA), and expressed as the mean ± S.E.M. of three experiments. The IC_50_ values were calculated by the EZ-Fit enzyme kinetics program (Perellela Scientific, Inc., Amherst, Mars, USA). GraphPad Prism 5, program was used for plotting dose dependant graphs of active plant extracts and for other graphs.

## Results

**Fig. 1:**
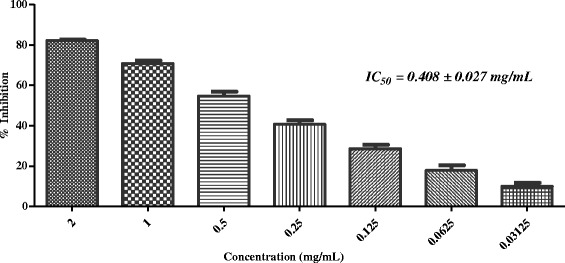
In-vitro anti-glycation activity of methanolic extract of *Glycyrrhiza glabra*

**Fig. 2:**
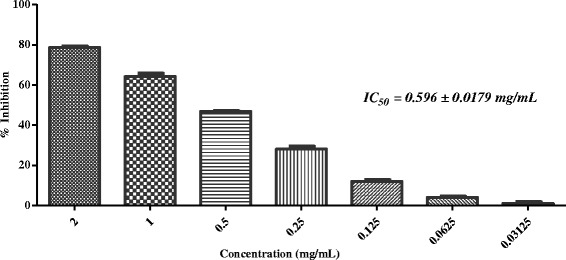
In-vitro anti-glycation activity of methanolic extract of *Rosa indica*

**Fig. 3:**
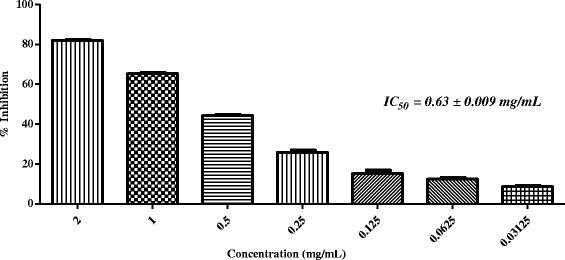
In-vitro anti-glycation activity of methanolic extract of *Sida cordifolia*

**Fig. 4:**
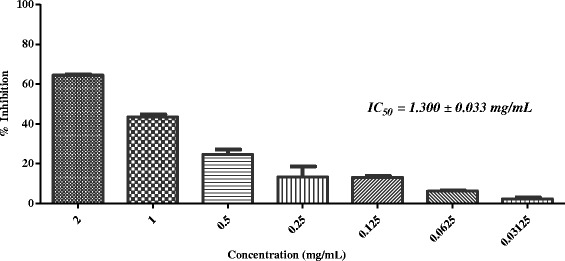
In-vitro anti-glycation activity of methanolic extract of *Plumbago zeylanica*

**Fig. 5:**
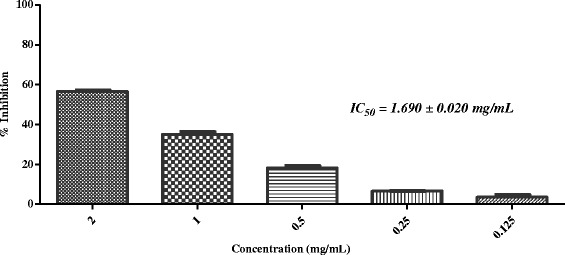
In-vitro anti-glycation activity of methanolic extract of *Tribulus terrestris*

Results revealed that out of 26 medicinal plants, five plants (*i.e. Sida cordifolia*, *Plumbago zeylanica*, *Tribulus terrestris*, *Glycyrrhiza glabra*, and *Rosa indica*) were able to inhibit the *in-vitro* protein glycation with IC_50_ values between 0.408- 1.690 mg/mL, while remaining plant extracts were found to be inactive as they showed less than 50 % inhibition at 2 mg/mL concentration (Table 1). Among five active plants, *Glycyrrhiza glabra* L. was found to be the most potent (IC_50_ = 0.408 ± 0.027 mg/mL; Fig. 1), followed by *Rosa indica* (IC_50_ = 0.596 ± 0.0179 mg/mL; Fig. 2) and *Sida cordifolia* L. (IC_50_ = 0.63 ± 0.009 mg/mL; Fig. 3). Extract of *Plumbago zeylanica* and *Tribulus terrestris* showed a week anti-glycation potential (IC_50_ = 1.300 ± 0.033 and 1.690 ± 0.020 mg/mL, respectively), as compared to other active plants in this study (Table 1; Figs. 4 and 5).

**Table 1:** Anti-glycation activity of extracts of medicinally important plants of Arabian origin

S. No.	Latin name	Family	Common English name	Part of the plant used	% Inhibition (at 2 mg/mL)	IC_50_ ± SEM (mg/mL)^1^
1.	*Abrus precatorius* L.	Fabaceae	Jequirity or Crab's eye	Fruits	5.03	NA^2^
2.	*Acacia concinna* (Willd.) DC.	Fabaceae	Soap-nut acacia	Fruits	−2.14	NA^2^
3.	*Azadirachta indica* A. Juss.	Meliaceae	Indian-lilac	Fruits	18.02	NA^2^
4.	*Balsamodendron mukul* Hook.	Burseraceae	Indian bdellium	Gum	−8.24	NA^2^
5.	*Calotropis procera* (Aiton) W. T. Aito.	Apocynaceae	Sodom apple	Flowers	5.70	NA^2^
6.	*Cassia senna* Linn.	Caesalpiniaceae	Senna	Leaves	42.89	NA^2^
7.	*Chenopodium album* L.	Amaranthaceae	Lamb's quarters	Whole plant	9.68	NA^2^
8.	*Citrullus colocynthis* (L.) Schrad.	Cucurbitaceae	Bitter apple	Fruits	−1.87	NA^2^
9.	*Daucus carota* L.	Apiaceae	Wild carrot	Seeds	13.26	NA^2^
10.	*Glycyrrhiza glabra* L*.*	Fabaceae	Licorice/ Liquorice	Roots	81.20	0.408 ± 0.027
11.	*Guilandina moringa* L.	Moringaceae	Moringa	Leaves	−29.72	NA^2^
12.	*Gymnema sylvestre* R. Br.	Apocynaceae	Miracle fruit	Leaves	39.28	NA^2^
13.	*Hedysarum alhagi* L.	Fabaceae	Camelthorn	Fruit peel	8.80	NA^2^
14.	*Lactuca sativa* L.	Asteraceae	Lettuce	Seeds	34.51	NA^2^
15.	*Plumbago zeylanica* L.	Plumbaginaceae	Ceylon leadwort or Doctor bush	Branches	64.53	1.300 ± 0.033
16.	*Punica granatum* L.	Lythraceae	Pomegranate	Flowers	0.293	NA^2^
17.	*Rhus coriaria* L.	Anacardiaceae	Sumac	Seeds	33.55	NA^2^
18.	*Rosa indica* L.	Rosaceae	Cyme rose	Flowers	78.56	0.596 ± 0.0179
19.	*Sesamum indicum* L.	Pedaliaceae	Sesame	Seeds	−103.80	NA^2^
20.	*Sida cordifolia* L.	Malvaceae	Country-mallow	Seeds	81.98	0.63 ± 0.009
21.	*Tamarindus indica* L.	Fabaceae	Tamrhindi	Fruits	−3.85	NA^2^
22.	*Tephrosia purpurea* (L.) Pers.	Fabaceae	Purple tephrosia	Branches	33.26	NA^2^
23.	*Trianthema pentandra* var. flava Blatt & Halb.	Aizoaceae	Trianthema	Roots	33.08	NA^2^
24.	*Tribulus terrestris* L.	Zygophyllaceae	Devil's thorn	Seeds	56.67	1.690 ± 0.020
25.	*Vitis vinifera* L.	Vitaceae	Wild grape	Fruits	2.14	NA^2^
26.	*Zizyphus vulgaris* Lam.	Rhamnaceae	Chinese date	Fruits	0.286	NA^2^
27.	Rutin^3^	–	–	–	95.56	0.196

^1^IC_50_ Values are presented in mg/mL ± standard error of mean of three assays; ^2^NA: Not Active; ^3^Standard inhibitor for anti-glycation assay

**Fig. 6:**
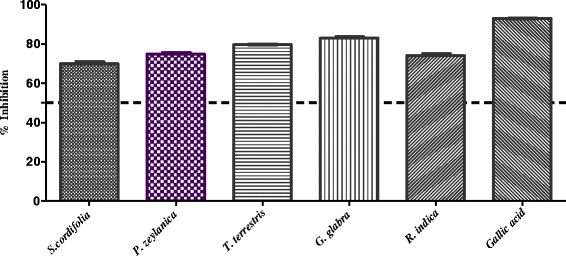
In-vitro antioxidant (DPPH) activity of medicinally important plants of Arabian origin which were found to be active against in-vitro anti-glycation assay

The plants which were found to be active against protein glycation in-vitro (*i.e. Sida cordifolia, Plumbago zeylanica*, *Tribulus terrestris*, *Glycyrrhiza glabra,* and *Rosa indica)* were evaluated for DPPH radical scavenging activity. Figure 6 shows that all plants were active (Table 2; Fig. 6). Gallic acid was used as positive control in this assay. Results revealed that *Sida cordifolia* L. showed a potent antioxidant activity when compared with remaining four active plants.

**Table 2:** In-vitro antioxidant activity of medicinal plants of Arabian origin (which were found to be active in BSA-MG glycation assay)

Scientific Name	DPPH radical scavenging activity		Iron Chelation activity	Superoxide anion radical scavenging activity	
	% RSA^a^	IC_50_ ± SEM^b^ (*m*g/mL)	% Chelation^a^	% RSA	IC_50_ ± SEM^b^ (*m*g/mL)
*Glycyrrhiza glabra* L*.*	82.99	0.237 ± 0.001	−9.50	5.00	–
*Plumbago zeylanica* L.	74.94	0.044 ± 0.001	−16.10	92.80	0.118 ± 0.004
*Rosa indica* L.	74.15	0.023 ± 0.0005	1.90	97.20	0.141 ± 0.003
*Sida cordifolia* L.	69.99	0.005 ± 0.0004	6.60	94.50	0.078 ± 0.002
*Tribulus terrestris* L.	79.69	0.157 ± 0.002	20.40	78.80	0.872 ± 0.011
Gallic acid^b^	93.13	0.004 ± 0.0004	–	–	–
EDTA^c^	–	–	99.10	–	–
Quercetin^d^	–	–	–	99.80	0.0318 ± 0.001

^a^ at 2 mg/mL concentration; IC_50_ Values are presented in mg/mL ± standard error of mean of three assays; ^b, c, d^ Standard inhibitors for antioxidant studies

**Fig. 7:**
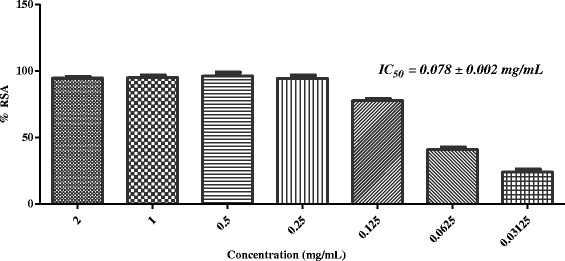
In-vitro superoxide anion radical scavenging activity of methanolic extract of *Sida cordifolia*

**Fig. 8:**
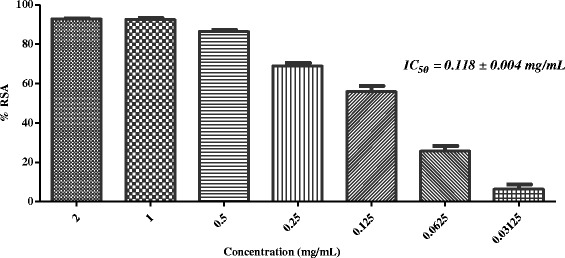
In-vitro superoxide anion radical scavenging activity of methanolic extract of *Plumbago zeylanica*

**Fig. 9:**
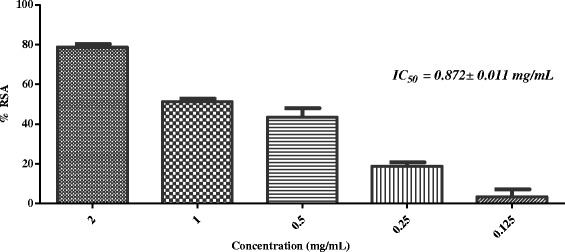
In-vitro superoxide anion radical scavenging activity of methanolic extract of *Tribulus terrestris*

**Fig. 10:**
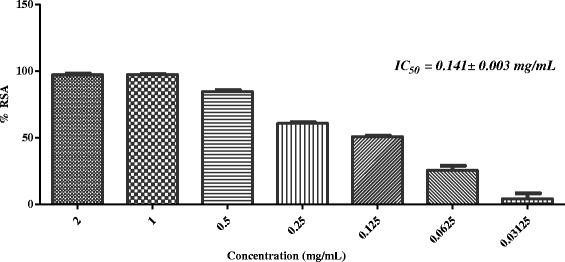
In-vitro superoxide anion radical scavenging activity of methanolic extract of *Rosa indica*

In the next stage, five active plants (*i.e. Sida cordifolia, Plumbago zeylanica*, *Tribulus terrestris*, *Glycyrrhiza glabra,* and *Rosa indica*) were evaluated for iron chelating ability. Table 2 showed that none of these has the ability to chelate with the iron at 2 mg/mL concentration. In the final step of this study, selected plants were evaluated for superoxide anion radical scavenging activity. Results showed that, except *G. glabra*, all other plants were found to scavenge the superoxide anion radicals effectively (Table 2, Figs. 7, 8, 9 and 10).

## Discussion

The present study was carried out to study medicinal plants of Arabian origin as anti-glycation agents. In this study, we systematically evaluated 26 medicinal plants for their anti-glycation activity potential (Table 1). Results revealed that out of 26 medicinal plants, five (*i.e. Sida cordifolia*, *Plumbago zeylanica*, *Tribulus terrestris*, *Glycyrrhiza glabra,* and *Rosa indica)* were found active against the *in-vitro* protein glycation. Among five active plants, *Glycyrrhiza glabra* L. (Voucher number: 37999) was found to be the most potent one. *G. glabra* belongs to the Fabaceae/Leguminosae family. It is famous for underground stems, which is widely used in flavor confectionery [21]. This plant is also known for its diverse biological activities, such as anti-inflammatory, anti-microbial, hepatoprotective properties. It is also used as folk remedy for sore throats, mouth ulcers, stomach ulcers, inflammatory stomach conditions, and indigestion [22–26]. *G. glabra* was also reported for hypoglycemic activity in rats [27]. *G. glabra-* based herbal formulations are known to exhibit anti-AGEs activities. Additionally a pure substance (glycyrrhizic acid) from the roots of this plant showed anti-glycation potential in high fat diet treated rats [14, 28, 29]. Major constituents of *G. glabra* include flavonoids, isoflavonoids, saponins, and tripentenes [30]. Literature has no report describing the in-vitro anti-glycation activity of root extracts of *G. glabra.* In our study methanolic extracts of *G. glabra* showed 63.42 % inhibition (IC_50_ = 0.408 ± 0.027 mg/mL) in BSA-MG glycation assay (Table 1; Fig. 1). Therefore in view of these results, *G. glabra* may be used as a therapeutic agent to reduce AGEs formation in diabetes.

*Rosa indica* L. ​(Voucher number: 91863) is an ornamental plant, known for perfuming effect. It possess pharmacological properties such as antioxidant, anti-fungal, anti-bacterial and urease inhibitory activities [31–33]. Manikandan et al. reported the synthesis of silver nanoparticles using extract of the petals of *Rosa indica* (ethanolic extract), and its in-vitro antibacterial, anticancer and anti-inflammatory activities. Different parts of *R. indica* (*e.g.* petals and buds) are known to treat runny nose, blocked bronchial tubes, asthma, and chest problems [34]. The bioactive compounds isolated, from *Rosa indica*, include flavonoids, alkaloids, phenols, saponins, and steroids [35]. There is no report describing the anti-glycation activity of *R. indica*. In our *in-vitro* experimental assay, *R. indica* showed a good anti-glycation potential with 78.56 % inhibition (IC_50_ = 0.596 ± 0.0179 mg/mL) (Table 1; Fig. 2).

The third most potent plant *Sida cordifolia* L. (Voucher number: 12135) belongs to Malvaceae family. Roots of *S. cordifolia* are used in coryza, pain, cardiac diseases, nervous disorders, and for anti-inflammatory, analgesic, hypoglycemic, antimicrobial, anti-hypercholesterolemic, antioxidant activities [36–40]. In addition, the extract of *S. cordifolia* has shown the anti-aging properties [41]. Major phytoconstituents of *Sida cordifolia* include alkaloids, flavonoids, steroids, phytoecdysteroids, and fatty acids [42]. As per literature survey, this is the first report describing the anti-glycation potential of the methanolic extracts of the seeds of *S. cordifolia*. In the present study, *S. cordifolia* showed 81.98 % inhibition of BSA-MG glycation with IC_50_ = 0.63 ± 0.009 mg/mL (Table 1; Fig. 3).

Crude extract of *Plumbago zeylanica* L. (voucher No. 24177) and *Tribulus terrestris* L. (voucher No. 53177) showed a week anti-glycation potential (IC_50_ = 1.300 ± 0.033, and 1.690 ± 0.020 mg/mL, respectively), when compared with the other active plants of this study (Table 1; Fig. 4 and 5).

The plant *P. zeylanica* showed many biological properties, such as anti-inflammatory, hypolipidimic, wound healing, antidiabetic, memory-inducing, blood coagulation, anti-malarial, anti-fertility, anti-microbial, anticancer, antiviral, antioxidant, and anti-larvicidal activities. The phytochemical investigation showed that these biological activities are due to the presence of compounds, such as elliptinone, zeylanone, sistosterol and plumbagin [43]. *Tribulus terrestris* L. is known for several pharmacological properties, and its use in folk medicine for the treatment of impotence, edema, rheumatism, kidney stones, and hypertension. *T. terrestris* contains phenols, saponins, alkaloids and sterols as active constituents [44].

Oxidative reactions are known to be involved in the protein glycation cascade. Most importantly, AGEs *via* their receptors (RAGEs), inactivate the enzymes and promote the formation of reactive oxygen species. Dual activity, *i.e.* antioxidant and anti-glycation, is therefore a valid approach for the treatment of complications resulting from hyperglycemia [11, 12]. The antioxidant activities of plants are mainly due to two mechanisms, *i.e.* scavenging the free radicals produced in the body or by chelating the transition metal [45]. Keeping this in view, all active plants (*i.e. Sida cordifolia, Plumbago zeylanica*, *Tribulus terrestris*, *Glycyrrhiza glabra,* and *Rosa indica)* were evaluated for their DPPH, superoxide anion radical scavenging and iron-chelating activities.

DPPH (2,2-diphenyl-1-picrylhydrazyl) is a stable free radical, in which electronic delocalization resulted in deep violet coloration. Certain plant extracts are able to donate hydrogen atoms and convert the DPPH radical into its reduced and stable form, and hence resulted in fading of violet color into pale yellow [45]. The in-vitro DPPH radical scavenging assay was performed with gallic acid as a positive control. Results revealed that all plants were active (Table 2; Fig. 6).

Glycation reaction, and AGEs are known to produce reactive oxygen intermediates (mainly superoxide anion and hydrogen peroxide) both in-vitro as well as in-vivo. In the in-vivo system, once generated, H_2_O_2_ can quickly enter inside the cell, while other activated oxygen species cannot. Within the cell, H_2_O_2_ can react with iron or copper in the Fenton reaction, and leads to the formation of hydroxyl radicals. These hydroxyl radicals contribute factors in diabetes-related oxidative stress [46]. Therefore metal chelators (*e.g.* Fe-chelators) can effectively serve as AGE-inhibitors. Interestingly when we evaluated the five active plants (*i.e. S. cordifolia, P. zeylanica*, *T. terrestris*, *G. glabra,* and *R. indica*) for iron chelating ability, all were found to be inactive, showing that they do not have ability to chelate with the iron at 2 mg/mL concentration.

Superoxide radical anion is formed from the reduction (*i.e.* one-electron) of free molecular oxygen by membrane-bound enzyme *i.e.* nicotinamide adenine dinucleotide phosphate Oxidase (NADPH). Ortwerth et al. reported that in glycation reaction, superoxide anion is formed by superoxide dismutase-dependent reduction of ferricytochrome C. They reported that Amadori products (formed from the reaction of lysine and small sugars) can generate superoxide anion even in the absence of metals [47]. Therefore, scavenging of superoxide anion was identifying as useful approach to inhibit the glycation mediated complications. In the final step of this study, we subjected all five plants for superoxide anion radical scavenging assay. Results showed that except *G. glabra*, all four plants scavenge the superoxide anion radicals effectively (Table 2, Figs. 7, 8, 9, and 10).

## Conclusions

In conclusion, the crude methanolic extracts of *Rosa indica* L., and *Sida cordifolia* L. have exhibited a potent protein anti-glycation activity in in-vitro BSA-MG anti-glycation model. On the basis of these findings, one of the possible mechanisms of their reported antidiabetic activities is the inhibition of glycation and antioxidant properties. The anti-glycation activity of these medicinal plants are in good agreement with their uses in antidiabetic herbal medicines. Therefore, these plants needs to be further investigated phytochemically as well as pharmacologically to identify the active constituents and to establish their therapeutic potential against glycation induced pathologies in diabetes.

## Abbreviations

AGEs, Advanced glycation endproducts; BSA, Bovine serum albumin; DM, Diabetes mellitus; DNA, Deoxyribonucleic acid; DPPH, 1,1-Diphenyl 2-picrylhydrazyl; em, Emission; ex, Excitation; H_2_O_2_: Hydrogen peroxide; IC_50_, Half maximal inhibitory concentration; MG, Methylglyoxal; RAGEs: Receptor for advanced glycation endproducts; ROS, Reactive oxygen species
